# Impulsivity is Associated with Increased Metabolism in the Fronto-Insular Network in Parkinson’s Disease

**DOI:** 10.3389/fnbeh.2015.00317

**Published:** 2015-11-30

**Authors:** Masoud Tahmasian, Luisa Rochhausen, Franziska Maier, Kim L. Williamson, Alexander Drzezga, Lars Timmermann, Thilo Van Eimeren, Carsten Eggers

**Affiliations:** ^1^Department of Neurology, University Hospital of CologneCologne, Germany; ^2^Department of Nuclear Medicine, University Hospital of CologneCologne, Germany; ^3^Sleep Disorders Research Center, Kermanshah University of Medical Sciences (KUMS)Kermanshah, Iran

**Keywords:** impulsive behaviors, FDG-PET, orbitofrontal cortex, anterior cingulate cortex, insula

## Abstract

Various neuroimaging studies demonstrated that the fronto-insular network is implicated in impulsive behavior. We compared glucose metabolism (as a proxy measure of neural activity) among 24 patients with Parkinson’s disease (PD) who presented with low or high levels of impulsivity based on the Barratt Impulsiveness Scale 11 (BIS) scores. Subjects underwent 18-fluorodeoxyglucose positron emission tomography (FDG-PET) and the voxel-wise group difference of FDG-metabolism was analyzed in Statistical Parametric Mapping (SPM8). Subsequently, we performed a partial correlation analysis between the FDG-metabolism and BIS scores, controlling for covariates (i.e., age, sex, severity of disease and levodopa equivalent daily doses). Voxel-wise group comparison revealed higher FDG-metabolism in the orbitofrontal cortex (OFC), anterior cingulate cortex (ACC), and right insula in patients with higher impulsivity scores. Moreover, there was a positive correlation between the FDG-metabolism and BIS scores. Our findings provide evidence that high impulsivity is associated with increased FDG-metabolism within the fronto-insular network in PD.

## Introduction

Impulsivity is an umbrella term that covers “actions that are poorly conceived, prematurely expressed, unduly risky, or inappropriate to the situation and that often result in undesirable outcomes” (Evenden, 1999). Impulsivity is generally considered as a personality trait that is associated with self-control deficiency and several problematic behaviors such as aggression, risk-seeking behavior, driving violation and also suicide attempt (Owsley et al., 2003; Fineberg et al., 2014; Gvion et al., 2014). Impulsive behaviors can be observed in healthy individuals (Chamorro et al., 2012), drug-dependent individuals (Ersche et al., 2011; Qiu et al., 2013) or patients with neuropsychiatric disorders including bipolar mood disorders, borderline personality disorder and attention-deficit/hyperactivity disorder (Nandagopal et al., 2011; Cackowski et al., 2014; Sebastian et al., 2014; Fossati et al., 2015). In addition, high impulsivity is a risk factor for impulse control disorders (ICDs) that consist of serious behavioral symptoms such as pathological gambling, compulsive shopping, binge eating and hyper sexuality. Crucially, ICDs may develop due to overstimulation of the mesolimbic system by dopaminergic medication (Lee et al., 2010; Voon et al., 2011a,b; Probst and Van Eimeren, 2013). ICDs are common non-motor symptoms in Parkinson’s disease (PD). For example, it has been shown that at least one form of ICDs was found in 13.6% of medicated PD patients (Weintraub et al., 2010). Moreover, the trait of impulsivity might be an important selection criterion for deep brain stimulation (DBS) of the subthalamic nucleus (STN), as DBS of the limbic part of the STN can reduce the activity of the inhibitory networks in PD (Jahanshahi, 2013). Thus, understanding the neural mechanisms of impulsivity in PD may lead to better treatment strategies in future.

Neuroimaging studies demonstrated that the fronto-insular network, including the orbitofrontal cortex (OFC), anterior cingulate cortex (ACC), medial prefrontal cortex (mPFC), dorsolateral prefrontal cortex (dlPFC), and insula, are involved in impulsive behavior in healthy controls (Matsuo et al., 2009a; Cho et al., 2013), patients with neuropsychiatric disorders (Antonucci et al., 2006; Matsuo et al., 2009b; Sebastian et al., 2014; Trost et al., 2014), high risk individuals for psychosis (Lee et al., 2013), and PD patients with ICDs (Cilia et al., 2008, 2011; Van Eimeren et al., 2010; Voon et al., 2011a; Biundo et al., 2015). In this study, we focused on impulsivity rather than ICDs to investigate the neural mechanisms underlying high level of impulsivity as a risk factor for developing ICDs in PD patients, while we controlled for age, gender, severity of disease, and levodopa equivalent daily dose (LEDD) for dopamine agonists. We hypothesized that PD patients with higher level of impulsivity have regional glucose metabolism alterations in the fronto-insular network, particularly in the OFC, ACC, and insula, which have been discussed to be associated with the inhibitory networks and impulsivity behaviors.

## Materials and Methods

### Subjects

Twenty-four right-handed patients (mean age 66.29, SD 6.01) with idiopathic PD were recruited from the outpatient clinic of the Department of Neurology, University Hospital of Cologne. The study was approved and registered by the medical ethics board of the University Hospital of Cologne in line with Human Research Committee guidelines. All subjects provided informed consent in accordance with the standard protocol approvals (Nr.10-278). On every subject, medical history and neurological examination were performed. Patients fulfilled criteria for PD based on the United Kingdom Parkinson’s Disease Society Brain Bank criteria (Hughes et al., 1992). Evaluation of motor symptoms was assessed with the Unified Parkinson’s Disease Rating Scale (UPDRS) Part III (Fahn et al., 1987; Van Hilten et al., 1994) and severity of PD was assessed with Hoehn and Yahr (1967) staging in both the ON- and the OFF-state. The OFF-state reflects withdrawal from dopamine replacement therapy for at least 12 h or from controlled-released drugs, such as dopamine agonists, for at least 72 h. The ON-state was defined as patient’s best response to 200 mg of levodopa after the OFF-state. Neuropsychological assessments were acquired in the regular daily medication ON-state. For subsequent analyses, the LEDD of dopamine agonists was calculated according to the guidelines of the German Neurological Society (Diener and Weimar, 2012). In addition, all patients were interviewed in a detailed survey that noted the side of onset and duration of disease since the first diagnoses. Two movement disorders specialists (C.E, L.T) assessed severity of PD. Our exclusion criteria were the following: (i) psychiatric comorbidities including depression (BDI-II score >19; Beck et al., 1996; Kühner et al., 2007), severe cognitive impairment or dementia (Mini Mental State *Exam* (MMSE) <27; Kessler et al., 2000); (ii) any other severe systemic diseases including cardiovascular diseases or diabetes mellitus; (iii) neurological diseases such as history of head trauma, stroke, brain tumor, epilepsy, or dyskinesia; and (iv) PD patients with diagnosis of ICDs.

### Neuropsychological Assessment

The Barratt Impulsiveness Scale (BIS) is a self-report questionnaire to evaluate impulsivity, which consists of 30 four-point Likert-type items reflecting frequency of occurrence. The scale was filled out by all patients during their regular daily medication. The BIS can be divided into three sub-scores including attention, motor, and non-planning impulsiveness. Higher BIS scores reflect higher level of impulsivity (Patton et al., 1995).

### Preprocessing and Analysis of FDG-PET Data

Preprocessing of fluorodeoxyglucose positron emission tomography (FDG-PET) images was carried out using Statistical Parametric Mapping (SPM8) (Wellcome Trust Center for Neuroimaging, London, UK) as described before Drzezga (2009), Eggers et al. (2014) and Tahmasian et al. (2015b). First, the scans were normalized to the standard stereotactical space using the standard PET template and then smoothed using a 6 mm full width at half maximum (FWHM) Gaussian filter.

### FDG-PET Data Acquisition

As described previously (Eggers et al., 2009), a high-resolution 24-detector ring PET scanner (ECAT EXACT HRRT, Siemens CTI, Knoxville, TN, USA) with 207 transaxial image planes and 1.219 mm voxel size was used in this study. Images were acquired with subjects in resting position, with background noise reduced, and with light dimmed. After the injection of 370 MBq of 18F-fluorodeoxyglucose (FDG), cerebral glucose metabolism was measured, reflecting the regional neural activity. Arterialized venous blood sampling allowed absolute quantification for all participants. The imaging was performed in the 3-D mode and was subsequently reconstructed as well as corrected for random artifacts, head motion, attenuation and scatter. The resolution of the reconstructed images was almost isotropic with 2.2 mm FWHM in the center and 2.5 mm FWHM at 10 cm off-axis. The FDG-PET measurement was performed with subjects in their regular medicated state (ON-state) to decrease head motion during scanning and to evaluate neural activity in a similar condition in terms of impulsivity in their daily routine.

### Statistical Analyses

Following our hypothesis, we divided our patients into two groups based on their BIS scores according to the published standards i.e., patients with higher impulsivity (*BIS* > 65, *n* = 8) and lower impulsivity (BIS ≤ 65, *n* = 16; Voon et al., 2007; Stanford et al., 2009). For group comparisons of demographic and neuropsychiatric data, we carried out two-sample *t*-tests as a parametric test applied on normally distributed data, and Mann-Whitney U-tests as a nonparametric test for not normally distributed data and also Fisher’s exact test for sex difference in the Statistical Package for Social Sciences, version 22 (SPSS). *P*-values less than 0.05 were considered statistically significant.

For group comparisons of FDG-PET data, a voxel-wise two-sample *t*-test in SPM8 was performed across the whole-brain, while PET images were normalized by the whole-brain FDG uptake values. The initial uncorrected threshold of 0.001 was applied for group comparison and results were reported as significant at *p*-value less than 0.05 with family-wise error (FWE) correction of the cluster-level. This analysis was controlled for covariates, including age, gender, severity of disease (UPDRS III OFF) and LEDD for dopamine agonists. We chose dopamine agonist LEDD instead of total LEDD because it has been shown that dopamine agonists change the activity of the OFC and rostral cingulate region in PD (Van Eimeren et al., 2009, 2010). Results with total LEDD as a covariate were highly similar (not shown). These analyses yielded a volume-of-interest (VOI) that showed significant metabolic changes in patients with higher impulsivity level compared to patients with lower impulsivity level. We chose the VOI based on significant metabolic changes in the whole brain voxel-wise group comparison to be independent from selection bias of *a-priori* defined regions. Subsequently, we extracted the absolute averaged FDG-metabolism within the mentioned VOI for each individual subject as applied previously in several neuroimaging studies (Matsuda et al., 2012; Tahmasian et al., 2013, 2015b; Wehrl et al., 2013; Klupp et al., 2014, 2015), then normalized those scores (FDG scores from the VOI divided to global uptake scores) and then performed independent *t*-test between groups using SPSS.

To detect the association between the FDG-metabolism and impulsivity, we performed a partial correlation analysis between the normalized averaged FDG-metabolism scores of the VOI and the total and sub-scores of BIS across all 24 patients in SPSS, controlling for covariates such as age, gender, severity of disease (UPDRS III OFF) and LEDD for dopamine agonists.

## Results

### Demographic and Neuropsychological Data

Our sample consisted of 24 non-demented, non-depressed, non-ICD PD patients. Demographic information is summarized in Table 1. The mean total BIS for the patients with low level of impulsivity was 53.18 (SD 7.60; range 41–63) and for the patients with a high level of impulsivity was 70.37 (SD 4.17; range 65–79). Between groups, there were significant differences on the total and sub-scores of BIS, including attention, motor and non-planning (*p* < 0.05, Table 1). Group comparisons demonstrated no significant differences regarding the severity of disease and severity of motor symptoms. However, there was a significant difference between LEDD calculated only for dopamine agonists as suggested previously (Tomlinson et al., 2010). Hence, we controlled for the effects of dopamine agonists in further analyses. One should note that there was trend towards a significant group difference regarding the LEDD-total and duration of disease since diagnosis.

**Table 1:** **Demographic and neuropsychological data (^a^ = Mann-Whitney-Test, ^b^ = *t*-test, ^c^ = Fisher’s exact test, degree of freedom was 22 for all group comparisons, results presented as mean ± SD)**.

	PD patients with lower impulsivity (*n* = 16)	PD patients with higher impulsivity (*n* = 8)	*p*-value	
Sex (female/male)	6/10	2/6	0.667^c^
Age (year)	65 ± 6.59	68.88 ± 3.75	0.14^b^
Duration since diagnosis	7.41 ± 4.23	11.38 ± 5.21	0.054^b^
Hoehn und Yahr OFF	3 ± 0.89	2.62 ± 0.51	0.349^a^
Hoehn und Yahr ON	2.5 ± 1.03	2.25 ± 0.46	0.588^a^
UPDRS III OFF	35.06 ± 14.17	29.50 ± 5.15	0.177^b^
UPDRS III ON	24.19 ± 13.09	21.38 ± 6.34	0.759^a^
LEDD—total (mg)	600.94 ± 356.45	956.12 ± 475.51	0.051^b^
LEDD—dopamine agonists (mg)	180.62 ± 133.85	318.62 ± 181.68	0.046^b^
MMSE	28.69 ± 1.138	28.75 ± 1.275	0.928^a^
BIS—total	53.18 ± 7.60	70.37 ± 4.17	0.000^b^
BIS—attention	14.37 ± 2.80	18.375 ± 2.38	0.002^b^
BIS—motor impulsivity	19.00 ± 3.01	22.625 ± 2.32	0.007^b^
BIS—non-planning	19.81 ± 5.39	29.5 ± 3.92	0.000^b^

*Abbreviations: BIS, barratt impulsiveness scale; LEDD, levodopa equivalent daily dose; MMSE, mini–mental state examination; PD, Parkinson’s disease; SD, standard deviation; UPDRS, unified Parkinson’s disease rating scale*.

### PD Patients with Higher Impulsivity Level Revealed Increased Glucose Metabolism in the Fronto-Insular Network

**Figure 1:**
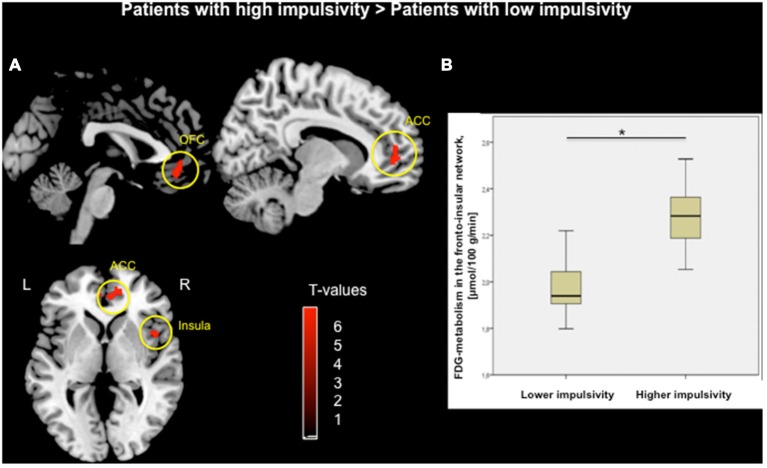
**(A)** Voxel-wise group comparison of FDG-metabolism generated by independent *t*-test in SPM8. Red maps illustrate increased metabolism in PD patients with higher impulsivity compared to PD patients with lower impulsivity in the orbitofrontal cortex (OFC), anterior cingulate cortex (ACC), and right insula (*p* < 0.05, FWE corrected in cluster level, bars represent range of *t*-values). **(B)** Group difference based on the averaged FDG-metabolism of the fronto-insular network including the OFC, ACC, and insula within (*p < 0.001).

Voxel-wise two-sample *t*-test between groups demonstrated higher metabolism within the fronto-insular network including the OFC, medial-frontal gyrus, ACC and insula, mainly on the right hemisphere (based on Automated Anatomical Labeling atlas; Tzourio-Mazoyer et al., 2002) in subjects with higher impulsivity level compared to individuals with lower level of impulsivity (*p* < 0.05, FWE corrected; Figure 1A, Table 2). Moreover, patients with higher impulsivity revealed higher averaged FDG-metabolism using the group difference VOIs determined by SPM8 (mean ± SD = 2.28 ± 0.14 vs. 1.97 ± 0.10; Figure 1B). On the other hand, PD patients with higher level of impulsivity had significant decreased FDG-metabolism in the superior parietal gyrus and occipital cortex compared to PD patients with lower level of impulsivity (*p* < 0.05, FWE corrected, not shown).

**Table 2:** **Voxel-wise group comparison *t*-test demonstrated increased metabolism in PD patients with higher impulsivity compared to PD patients with lower impulsivity**.

Anatomical region	L/R	Cluster	*p*-value (FWE-corrected)	*T*-score	Peak coordinates (MNI)
Orbitofrontal cortex	L	170	0.004	5.82	−2, 38, −10
Medial frontal gyrus	R	170	0.004	5.77	12, 48, 2
Anterior cingulate cortex	R	170	0.004	4.47	4, 44, 0
Insula-operculum	R	115	0.029	5.24	46, 2, 14
Insula	R	115	0.029	4.75	46, 10, 2

*Abbreviations: FWE, family-wise error; MNI, Montreal Neurological Institute*.

### Association between Impulsivity and Glucose Metabolism in the Fronto-Insular Network

**Figure 2:**
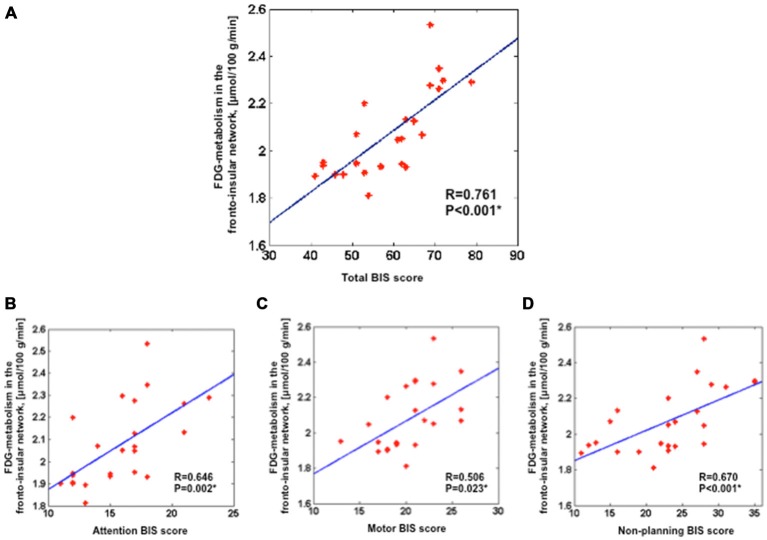
**Positive correlation between the averaged FDG-metabolism within the group comparison volume-of-interest (i.e., the fronto-insular network) extracted for each subject and the total BIS scores (A), attention BIS score (B), motor BIS score (C), non-planning BIS score (D).** Partial correlations were performed across all 24 PD patients with additional covariates such as age, sex, severity of disease and LEDD for dopamine agonists; *p < 0.05). BIS, Barratt Impulsiveness Scale; LEDD, levodopa equivalent daily dose.

We assessed the link between FDG-metabolism of the fronto-insular network and BIS scores. Results showed a positive correlation between the averaged FDG-metabolism and total BIS scores (*r* = 0.761, *p* < 0.001) across all patients (Figure 2A). Furthermore, significant positive correlations were found between the FDG-metabolism and BIS sub-score for attention (*r* = 0.646, *p* < 0.05), motor (*r* = 0.506, *p* < 0.05), and non-planning impulsivity (*r* = 0.670, *p* < 0.001; Figures 2B–D).

## Discussion

To assess the neural correlates of impulsivity in PD patients, we compared glucose metabolism of patients with higher impulsivity level and patients with lower impulsivity level. We found that patients with higher impulsivity level showed increased glucose metabolism within the fronto-insular network including the OFC, medial frontal gyrus, ACC, and right insula (Figure 1). Moreover, our findings demonstrated positive correlations between the averaged FDG-metabolism of those regions and BIS scores (i.e., total and sub-score for attention, motor, and non-planning impulsivity) across all patients (Figure 2). These results provide further evidence that higher impulsivity is linked with altered function of the fronto-insular network. Our findings are in line with previous reports indicating that high impulsivity is associated with structural and functional changes of regions associated with reward-related decision making and impulse control behavior including the OFC and ACC in healthy controls and individuals at ultra-high risk for psychosis (Horn et al., 2003; Brown et al., 2006; Cilia et al., 2008; Matsuo et al., 2009a; Cho et al., 2013; Lee et al., 2013). PD patients with higher level of impulsivity also demonstrated lower FDG-metabolism in the superior parietal gyrus and occipital cortex compared to other group. Based on our hypothesis we did not expect changes in these parieto-occipital regions. These findings might be additionally due to the increased FDG-metabolism in the fronto-insular network a secondary compensatory change of network activity.

Its worthy to note that FDG-PET imaging provides a quantitative measurement of regional metabolism within the synaptic terminals of the neuron-astrocyte functional unit. In detail, after injection of FDG, its tissue uptake increases in the active region, which correlates with the local metabolism of brain tissue. Hence, increase of glucose uptake provides indirect evidence of higher synaptic metabolism in a particular region (Lucignani and Nobili, 2010).

### Neural Correlates of Impulsivity within the Fronto-Insular Network

Distinct brain regions are responsible for processing of reward-related learning, goal-directed actions, decision-making and the formation of habits (Schultz et al., 2000; Torregrossa et al., 2008). Among them, the OFC is involved in sensory and emotional integration, encoding the affective value of reinforcers and evaluation of the expected rewards/punishments of a decision. Therefore, the OFC has an important role in adaptive decision-making, guiding behavior, judgments, and behavioral regulation (Kringelbach, 2005; Torregrossa et al., 2008; Schoenbaum et al., 2011). Animal studies revealed that OFC lesions result in failure to assess the value of an outcome under changing conditions, improper inhibition of motor responses, devalue the reinforce, increase in habitual responding and increased premature responses (Torregrossa et al., 2008). Thus, it seems that activity of the OFC is essential for proper impulse control. Moreover, it has been shown that the association of the lateral PFC to both aggression and attentional impulsivity depends on OFC contribution (Gansler et al., 2011). In a functional neuroimaging (fMRI) study, Horn et al. (2003) applied Go/No-Go task. This task is often used to assess a participant’s ability to sustain attention and inhibit responses. The authors demonstrated a significant activation in the anterior lateral OFC during the inhibition task. They showed that subjects with higher impulsivity activated the right inferior frontal gyrus, posterior lateral OFC and anterior insula (Horn et al., 2003). Similarly, our results support the idea that hyperactivity of the OFC is essential for proper inhibition in PD patients with high impulsivity.

The OFC has reciprocal connections with the ACC, which is involved in executive functions such as attention, inhibition and emotion regulation (Devinsky et al., 1995; Banks et al., 2007; Rushworth et al., 2007). Another fMRI study demonstrated that activation of several regions, including the ACC, was positively correlated with impulsivity level during inhibitory control paradigms, suggesting a regulatory role of ACC in modulating impulsive behaviors (Brown et al., 2006). Recently, Kerr et al. (2015) revealed that impulsivity was linked with higher activation of the ACC and amygdala during anticipation of the primary reward. In addition, impulsivity was negatively associated with functional connectivity between the ACC and amygdala (Kerr et al., 2015). Wilbertz et al. (2014) highlighted a link between urgency as an impulsivity subdomain and a network including the inferior frontal gyrus, anterior insula and dorsal ACC. Another Go/No-Go fMRI study revealed that subjects with internet gaming addiction had hyperactivity during No-Go trials in several brain regions including the left superior medial frontal gyrus, right ACC, right superior/middle frontal gyrus. Interestingly, activation of the superior medial frontal gyrus was positively associated with BIS-11, suggesting an association between impulsivity and impaired prefrontal impulse inhibition (Ding et al., 2014). Moreover, it has been reported that self-control and successful inhibition of impulsive behaviors, particularly motor impulsivity, and reactive aggression depends on the anterior insula activity (Dambacher et al., 2015).

Several studies highlighted the relationship between gray matter volume changes and the BIS scores in healthy controls and subjects at ultra-high risk for psychosis (Matsuo et al., 2009a; Cho et al., 2013; Lee et al., 2013). For example, Cho et al. (2013) found positive correlations between volume of mPFC, dlPFC, OFC, ACC and total, non-planning, and attention/cognitive BIS scores but not with motor impulsivity. Churchwell and Yurgelun-Todd (2013) found a positive linear association between anterior insula thickness and non-planning imulsivity, and both of them had negative correlations with age. Similarly, it has been demonstrated that patients with major depressive disorder, alcoholism, posttraumatic stress disorder, attention-deficit/hyperactivity disorder, antisocial personality disorder or bipolar disorder showed positive correlations between the left, right, and total OFC gray matter volume and BIS motor impulsivity scores and aggression (Antonucci et al., 2006). Our results are similar to these findings, as we found significant positive associations between the FDG-metabolism of the fronto-insular network and the total, non-planning, attention and motor impulsivity. On the other hand, Matsuo et al. (2009a) demonstrated that gray matter volumes of the bilateral OFC and left ACC were negatively correlated with the total BIS scores. More specifically, they found negative associations between the right OFC volume and non-planning impulsivity, and between the left OFC volume and motor impulsivity.

In the right fronto-insular cortex and anterior limbic area of the human brain there are large bipolar neurons so-called “von Economo neurons”, particularly in the ACC and the anterior insula. These neurons are involved in empathy, social awareness, and self-control and their numbers are reduced in several neuropsychiatric disorders including fronto-temporal dementia, schizophrenia, bipolar disorder, addiction and ICDs (Allman et al., 2011a,b; Kim et al., 2012).

Taken together, the above-mentioned studies showed that the fronto-insular network is critically involved in impulsiveness. With regards to our findings of the increased metabolism within this network, it is possible to speculate that subjects with higher impulsivity scores need this network to be more active in order to inhibit their impulses, compared to subjects with lower impulsivity.

### Impulsivity vs. Impulse Control Disorders

Although impulsivity is a natural behavior that can be controlled by inhibitory mechanisms in healthy individuals, it can be considered as a risk factor for ICDs in patients with PD (Cilia and Van Eimeren, 2011; Probst and Van Eimeren, 2013). Patients with ICDs such as pathological gambling, hypersexuality, and kleptomania have more compulsive characteristics resulting in failure to resist aggressive impulses (Weiss and Marsh, 2012; Fineberg et al., 2014). Although the neural systems for regulating impulsive, compulsive, and habitual behaviors have an overlapping regional pathophysiology (i.e., activation of the OFC), impulsivity and subdomains of ICDs have different pathophysiological mechanisms (Torregrossa et al., 2008; Leeman and Potenza, 2012). Accordingly, one should be aware that impulsivity and ICDs are conceptually and pathophysiologically distinct.

A recent study demonstrated that PD patients with ICDs had cortical thinning in fronto-striatal circuitry including the right superior OFC, left rostral middle frontal, bilateral caudal middle frontal region, corpus callosum, right accumbens, as well as an increase in the left amygdala. Moreover, they found a positive correlation between severity of impulsive symptoms and cortical thickness of left rostral middle frontal, inferior parietal, and supramarginal regions (Biundo et al., 2015).

Several fMRI studies revealed that dopamine agonist therapy mediates the ability of PD patients to control their impulses and may lead to high impulsivity and ICDs (Cilia et al., 2008; Van Eimeren et al., 2009, 2010; Ray and Strafella, 2010; Voon et al., 2011a; Weiss and Marsh, 2012; Napier et al., 2015). For example, it has been suggested that PD patients with pathological gambling have an ACC-striatal disconnection and also a hyperactivity in the OFC, hippocampus, amygdala, insula, and ventral pallidum, possibly associated with a drug-induced overstimulation of relatively preserved reward-related neuronal systems (Cilia et al., 2008, 2011). Van Eimeren et al. (2010) demonstrated that in the lateral OFC, rostral cingulate zone, amygdala, and external pallidum, healthy controls had higher activity in response to dopamine agonist, while PD patients with pathological gambling showed a significant DA-induced reduction of activity. To control for the influence of dopamine agonists on the suggested relationship (Tahmasian et al., 2015a), we applied a partial correlation approach, which accounts separately for influences of medication (LEDD for dopamine agonists) on local FDG-metabolism. Furthermore, the results were also independent from age and sex.

Due to our results, we assume that higher activity of fronto-insular network is necessary in patients with higher impulsivity. In particular, patients with higher impulsivity level have self-control deficiency and tendency to do problematic risky behaviors (Owsley et al., 2003; Fineberg et al., 2014; Gvion et al., 2014). Thus, they need to inhibit their impulses more than subjects with lower impulsivity level. As mentioned above, patients with lesions or atrophy in the OFC and ACC show more impulsive, antisocial, and risky behaviors (Winstanley et al., 2004; Berlin et al., 2005; Matsuo et al., 2009a,b; Kerr et al., 2015). Taken together, we suggest that the observed increased activity in the inhibitory network (Van Eimeren et al., 2010) within the fronto-insular regions is necessary to allow an active inhibition of risk-related impulsive behaviors, particularly in patients with higher impulsivity (for review, see Seguin, 2004; Crews and Boettiger, 2009; Perry et al., 2011). One should note that our subjects had higher impulsivity and not ICDs diagnosis. Hence, future studies should systematically compare subjects with different levels of impulsivity vs. healthy subjects and patients with ICDs to provide explicit proof of this hypothesis.

## Limitations

Our study has several limitations: (i) albeit the BIS scale is the most common tool to assess of impulsivity, it is a self-report and subjective questionnaire. Thus, it is not an objective assessment of impulsivity; (ii) it should be noted that our findings may be limited to moderate impulsivity level of our subjects and can not be generalized to ICDs in PD patients; (iii) the observed difference in glucose metabolism might be due to underlying mechanism including the different levels of von Economo neurons in the fronto-insular network, receptor availability or genetic difference across subjects but these data were not available for us to correct for their influence. In particular, gray matter volume difference is probably one of the most important confounding factors, but we did not have structural MRI data from these subjects to perform atrophy correction; and (iv) our sample size was rather small, particularly in patients with higher impulsivity level, we also did not include healthy control subjects or PD patients with ICDs. Future studies with larger sample size should consider atrophy correction and systematically compare healthy controls and PD patients with and without ICDs.

## Conclusion

In summary, the current study provides evidence that PD patients with higher impulsivity level have increased glucose metabolism within the fronto-insular network compared to PD patients with lower impulsivity level. The data are consistent with several structural and fMRI studies, suggesting that the activity of fronto-insular network is essential for proper impulse inhibition, particularly in PD patients with higher impulsivity. Our findings shed new light on the neural correlates of impulsivity in PD.

## Conflict of Interest Statement

The authors declare that the research was conducted in the absence of any commercial or financial relationships that could be construed as a potential conflict of interest.
